# Retinal Microperimetry as a Novel Tool for Early Detection of Subclinical Cognitive Dysfunction and Brain Damage in Type 1 Diabetes: A Pilot Study

**DOI:** 10.1002/edm2.70018

**Published:** 2024-12-21

**Authors:** Manel Mateu‐Salat, Nicole Stanton‐Yonge, Frederic Sampedro Santaló, José Ignacio Vela, Jesús Díaz Cascajosa, Eva Safont Pérez, Daniela Rego‐Lorca, Ana Chico

**Affiliations:** ^1^ Institut de Recerca Sant Pau (IR Sant Pau) Barcelona Spain; ^2^ Endocrinology and Nutrition Unit Hospital General de Granollers Granollers Spain; ^3^ Imaging Diagnostic Department Hospital de la Santa Creu i Sant Pau Barcelona Spain; ^4^ Ophthalmology Department Hospital de la Santa Creu i Sant Pau Barcelona Spain; ^5^ Endocrinology and Nutrition Department Hospital de la Santa Creu i Sant Pau Barcelona Spain; ^6^ CIBER‐BBN, ISCIII Madrid Spain; ^7^ Faculty of Medicine Universitat Autònoma de Barcelona Barcelona Spain

**Keywords:** brain damage, cognitive dysfunction, hypoglycaemia, microperimetry, type 1 diabetes

## Abstract

**Context:**

Retinal microperimetry (MPR) is a non‐invasive method that measures retinal light sensitivity (RS) and gaze fixation stability (GFS). MPR has been described as a marker of cognitive impairment in people with Type 2 diabetes, but it has never been assessed in people with Type 1 diabetes (T1D). Our group described subclinical cognitive alterations, structural brain differences, and increased levels of light chain neurofilament (NfL) in people with T1D and impaired awareness of hypoglycaemia.

**Objective:**

To measure RS and GFS using MPR in individuals with T1D and evaluate its correlation with neuropsychological assessment, plasma NfL levels and CGM‐derived glucometric parameters. Secondary objectives: to evaluate the possible differences of RS and GFS in people with T1D depending on hypoglycaemia awareness.

**Design, Setting and Participants:**

Pilot observational study, people with T1D without clinical cognitive impairment, moderate–severe retinopathy or glaucoma. MPR was performed with MAIA3.

**Results:**

A total of 30 subjects were studied: 40% women, age 58 ± 11 years; T1D duration 31 ± 9 years, mild retinopathy 33%. RS was 27.5 dB (26.1–28.3) and GFS(%) 97.6% (93.5%–99.5%). We found a correlation between RS and memory alteration tests (*p* = 0.016) and between GFS(%) and a composite of attention and executive neuropsychological tests (*p* = 0.025). An inverse correlation between GFS and time below range was found. No correlation was found with NfL.

**Conclusion:**

This first exploratory study in people with T1D supports the potential utility of MPR as a screening tool for subclinical neurocognitive alterations in this population.

## Graphical abstract

Retinal microperimetry is a non‐invasive method described as a marker of cognitive dysfunction in type 2 diabetes but not type 1 (T1D). Aim: to assess whether retinal sensitivity (RS) and gaze fixation stability (GFS) correlate with neuropsychological tests, light‐chain neurofilament (NfL) and glucometris parameters in people with T1D (*n* = 30). Results: significant correlations between – RS & memory alteration test (positive) – GFS & executive & attention tests – Fig 1 (positive) – GFS & time of glucose below range (TBR) – Fig 2 (inverse).

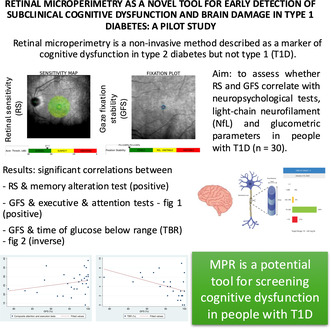

## 1 Introduction

Type 1 diabetes (T1D) is a chronic disease that affects various body systems, including the nervous system. People with T1D are at an increased risk of developing cognitive dysfunction compared to the general population [1, 2]. This risk is attributed to several mechanisms, including chronic hyperglycaemia, angiopathy, neuroinflammation and cellular brain damage. Moreover, severe hypoglycaemia can also play a fundamental role in the development of cognitive dysfunction. Impaired awareness of hypoglycaemia (IAH), defined as the inability to sense the onset of hypoglycaemic events, increases the risk for recurrent and severe hypoglycaemia and that may further increase the risk of neurological damage [3, 4, 5, 6, 7]. Our group has described subclinical cognitive alterations [8], structural cerebral differences (in both grey and white matter) [9] and increased plasma NfL levels (a biomarker for brain damage) [10] in patients with T1D with IAH (T1D‐IAH). Mainly, patients with T1D‐IAH have shown worse performance in a task measuring processing speed and attention (trail making test A) and neuropsychological differences using event‐related potentials (ERPs), suggesting that IAH is associated with disrupted neuronal processes [8]. Using brain magnetic resonance imaging (MRI) we also detected that patients with T1D‐IAH had reduced grey matter (GM) volumes and cortical surface areas, especially in frontal and parietal regions, as well as reduced fractional anisotropy (FA) values in major white matter (WM) tracts, which correlated with both episodes of severe hypoglycaemia and IAH severity [9]. Finally, we assessed plasma neurofilament light chain (NfL) levels, a recognised biomarker for neurodegeneration [11] associated to neuronal loss and axonal damage, finding higher levels in patients with T1D‐IAH, which correlated to reduced cerebral GM volume and IAH severity [10]. The detection of neurodegeneration through the described techniques requires advanced and costly tools, trained personnel and a substantial amount of time. Thus, the development of more affordable and user‐friendly techniques for the detection of neurodegeneration is necessary to improve the early identification of this condition and to increase the availability of effective interventions.

Microperimetry (MPR) is a diagnostic tool that measures retinal sensitivity (RS) by measuring the light sensitivity of specific points in the retina assessing the minimal amount of light needed to stimulate visual response in each tested location. Moreover, it provides information on gaze fixation stability (GFS) [12]. The retina is a brain‐derived tissue and, as such, it has been suggested that its alterations can correlate to brain damage, providing a non‐invasive way of assessing neurodegeneration [13, 14, 15]. MPR can detect subtle changes in retinal function before structural and visible signs of damage appear and it has been proposed to be a useful tool for detecting and monitoring neurodegeneration in many diseases, including diabetes. This method is non‐invasive, repeatable, can be performed independently of the education level, and can provide detailed information about the specific areas of the retina that are affected by disease. It has been described that the combination of RS and GFS correlate with brain imaging findings (MRI and PET) and can identify patients with mild cognitive impairment and dementia in people with Type 2 diabetes (T2D) [14, 15] as well as people with obesity and insulin‐resistance [16]. To the best of our knowledge, the utility of MPR as a marker of neurodegeneration has not been described in T1D.

Our study aims to conduct a first exploratory analysis of the potential of using MPR as a tool for early detection of cognitive dysfunction in patients with T1D. Given the distinct pathogenesis, clinical course, and target population of T1D compared to T2D, it is imperative to examine its applicability in this specific population. If the findings are positive, larger studies could be conducted to determine whether this non‐invasive and cost‐effective technology could be implemented as a routine tool for monitoring cognitive function in T1D patients, thereby improving patient care and outcomes. Early detection of cognitive impairment in T1D patients using MPR could facilitate timely interventions, leading to improved patient outcomes and a better quality of life. By shedding light on the potential of MPR in T1D patients, our study could open a door to how clinicians diagnose and monitor cognitive function in this population.

Continuous interstitial glucose monitoring (CGM) has proven to improve glucose control and to reduce recurrent and severe hypoglycaemia events, especially in people with T1D‐IAH who are at the highest risk [17]. Since the use of CGM has become widely available in our population in the last years, we also studied the correlation between CGM‐derived glucometric parameters with MPR outcomes.

A previous version of this manuscript was uploaded as a preprint in Research Square [18].

## 2 Hypothesis

Main hypothesis: MPR could be a useful tool for the early detection of subclinical cognitive dysfunction and brain damage in people with T1D with and without IAH.

Secondary hypothesis: MPR findings correlate with those related to cognitive dysfunction and brain damage such as: neuropsychological alterations, plasma NfL levels and glucometric parameters, mainly time below range (TBR).

## 3 Objectives

Main objective: to measure RS and GFS using MPR in individuals with T1D and evaluate its correlation with neuropsychological assessment, plasma NfL levels and CGM‐derived glucometric parameters.

Secondary objectives: to evaluate the possible differences of RS and GFS in people with T1D depending on hypoglycaemia awareness (IAH vs. NAH).

## 4 Methods

### 4.1 Sample and Assessments

This is an observational study carried out using a sample from previous studies [8, 9, 10]. Recruitment was consecutive and based on a case–control investigation of people with T1D and IAH (cases) and NAH (controls). Patients were re‐recruited for the present study. Inclusion criteria were as follows: individuals with T1D, follow‐up at the Department of Endocrinology and Nutrition at Hospital de la Santa Creu i Sant Pau, age 18 years or older and duration of T1D for a minimum of 5 years. Individuals with previously diagnosed cognitive impairment were excluded in the initial recruitment. All individuals had a mini mental state examination (MMSE) score equal or above 27 (considered normal), otherwise, those with an altered MMSE would have been excluded. In this specific study we also excluded those with moderate to severe retinopathy [19] and glaucoma, as these conditions independently affect the outcome of the MPR.

Hypoglycaemia awareness was assessed by the Clarke questionnaire [20] to classify the participants as having IAH (Clarke Score 4 or more) or normal awareness to hypoglycaemia (NAH) (Clarke Score 2 or less). Individuals with a Clarke score equal to three (indeterminant) were excluded.

Clinical data were collected from hospital records. Cognitive performance by neuropsychological tests and plasma NfL levels were obtained as per previous studies [8, 9, 21]. The neuropsychological tests battery included MMSE as screening, memory alteration test (TAM), the word learning subtest of the Consortium to Establish a Registry for Alzheimer's Disease (CERAD) which included CERAD word list memory, recall and recognition; trail making test A and B (TMT‐A and TMT‐B), phonetic verbal fluency, semantic verbal fluency and Boston naming test. Raw scores were adjusted and classified qualitatively using the normative data for Spanish population (NEURONORMA project) [22] for TMT‐A, TMT‐B, verbal fluency tests and Boston naming test. A composite of attention and executive function related tests (TMT‐A, TMT‐B and phonemic verbal fluency) was calculated adding the corresponding adjusted scores which use the same scale [22]. All tests were administered in Spanish by the same neuropsychologist, and all participants were native speakers.

NfL levels were measured in plasma with the Simoa Human NF‐light Advantage kit using the Single Molecule Array technology (Simoa; Quanterix) [23] in a SR‐X Biomarker detection system by following the manufacturer's instructions.

### 4.2 Microperimetry Assessment

MPR was performed by the same trained optometrist using the Macular Integrity Assessment device (MAIA 3, Topcon) in scotopic conditions and without pupillary dilatation, under normoglycemic conditions and without recent severe hypoglycaemia. Retinal sensitivity (RS) in decibels (dB) was evaluated at 37 macular points, 1 point centred on the fovea and 36 points spread over three rings of 12 points. Gaze fixation stability (GFS) and macular integrity (MI) were also automatically measured and recorded. Right eye was first examined. The same procedure was executed in all cases. Duration of the test was around 20 min. MAIA measures RS through MPR, which assesses the minimum light intensity that patients can perceive when stimulated with spots of light. The examination can be customised and covers a 10° diameter area with 37 measurement points, using a white LED and a 36 dB dynamic range. The results are expressed in decibels (dB) where 0 corresponds to no sensitivity and 36 dB to the best sensitivity according to the device used. The dB scale is classified according to the MAIA normative studies into three categories: abnormal, suspect, and normal [12]. In our group, the thresholds between abnormal and suspect were between 22 and 24 dB, and between suspect and normal, between 24 and 27 dB.

MAIA assesses GFS by tracking eye movements and plotting the distribution of fixation points, identifying two main points which are used to calculate the centre of the stimuli grid and the stability of fixation, respectively. GFS measures how steadily a patient can fixate on a target, with P1 indicating the percentage of fixation points within 1° of the target and P2 the percentage within 2°, both reflecting the accuracy and steadiness of fixation. The classification of stability is based on the following criteria: if more than 75% of the fixation points are located within P1, the fixation is classified as “stable”; if less than 75% of the fixation points are located within P1, but more than 75% of the fixation points are located within P2, the fixation is classified as “relatively unstable”; lastly, if less than 75% are located within P2, the fixation is classified as “unstable” [12]. Moreover, it provides with the Bivariate Contour Ellipse Areas (BCEA) which quantifies fixation stability by fitting an ellipse around recorded fixation points. BCEA63 represents the area that contains 63% of the fixation points, providing a measure of central fixation stability, while BCEA95 includes 95% of the points, capturing a broader range of eye movements. These metrics are useful for assessing how well subjects can maintain their gaze on a target. To sum up, GFS refers to how consistently a person can maintain their gaze on a target, while BCEA measures the spread of fixation points. Thus, a higher GFS means better fixation stability, while a larger BCEA demonstrates a lower fixation stability.

To assess reliability of the test, we considered fixation losses, planning to exclude those tests with fixation losses > 25%.

For analysis purposes, we calculated average RS (dB), the average of P1 and P2 (%), BCEA63 and BCEA95 for both eyes for each patient. The average of P1 and P2 (%) will be referred to as GFS(%).

### 4.3 CGM‐Derived Glucometric Assessment

CGM‐derived glucometric data were obtained for the 90 days prior to MPR assessment. Patients not using CGM or with a time of use below 80% were excluded from this analysis. Times in range were classified according to Battellino et al. [24]: time in range (TIR) as 70–180 mg/dL, time above range (TAR) as > 180 mg/dL and time below range (TBR) as < 70 mg/dL.

### 4.4 Statistical Analyses

Statistical analysis was performed using STATA MP14. Variables are expressed as mean ± standard deviation or median (p25–p75) depending on their distribution nature, as SR and GFS(%) showed an asymmetrical distribution, even though mean and median did not present major differences. Comparisons of variables between groups were performed using the Mann–Whitney (Wilcoxon rank‐sum) or Fisher exact tests. Correlations between RS and GFS(%) with other quantitative variables such as MRI metrics and NfL levels were performed using the Spearman test. To adjust for covariables when needed, we performed a multiple regression analysis. Prior to performing the multiple regression analysis, we performed a Spearman test and Pearson correlation to assess the reliability of the parametrical approximation in our sample, which did not show major differences.

## 5 Results

The initial sample consisted of 40 participants. Of these, two were excluded from the study as they were unable to attend the hospital for testing. An additional eight participants were excluded due to moderate to severe retinopathy and/or glaucoma (two individuals with moderate retinopathy, two with proliferative retinopathy, three with both proliferative retinopathy and glaucoma, and one with glaucoma). Finally, 30 people with T1D were studied. Clinical characteristics and MPR results (RS and GFS) are shown in Table 1. No patients needed to be excluded for presenting fixation losses over 25%.

**TABLE 1:** Clinical characteristics of the subjects included, and microperimetry results.

*n*	30
Age (years)	58.26 ± 10.56
Sex (% females)	40
Education (years)	14.43 ± 5.69
T1D duration (years)	31.33 ± 9.27
Type of insulin therapy (%)	
Multiple daily injections	73.33
Insulin pump	26.67
Severe hypoglycaemia episodes last 5 years	1.06 ± 2.01
Retinopathy (%)	
No	66.67
Mild	33.33
Hypertension (%)	43.33
Dyslipidemia (%)	60
Smoking (%)	
No	36.67
Previous	33.33
Current	30
Retinal sensitivity (dB) (median and IQR)	
Right eye	27.85 (26.5–28.5)
Left eye	27.5 (26.4–28.4)
Average	27.48 (26.1–28.3)
Right eye (%)	
Normal	86.67
Suspect	3.33
Abnormal	10
Left eye (%)	
Normal	93.10
Suspect	6.90
Abnormal	0
Gaze fixation stability	
P1 (%) (median and IQR)	
Right eye	95 (88–98)
Left eye	99.5 (97–100)
Average	95 (88–98)
P2 (%) (median and IQR)	
Right eye	99.5 (97–100)
Left eye	100 (99–100)
Average	99.5 (97.5–100)
GFS(%) (%) (median and IQR)	
Right eye	97 (92.5–99)
Left eye	98.5 (97.5–99.5)
Average	97.63 (93.5–99.5)
BCEA63 (deg^2^) (median and IQR)	
Right eye	0.8 (0.4–1.6)
Left eye	0.5 (0.3–0.9)
Average	0.68 (0.35–1.4)
BCEA95 (deg^2^) (median and IQR)	
Right eye	2.3 (1.1–4.8)
Left eye	1.5 (0.8–2.8)
Average	2 (1.05–4.15)
Qualitative stability right eye (%)	
Stable	90
Relatively unstable	10
Unstable	0
Qualitative stability left eye (%)	
Stable	89.66
Relatively unstable	10.34
Unstable	0

Abbreviations: IQR: interquartile range; T1D: Type 1 diabetes.

### 5.1 Hypoglycaemia Perception

Half of the patients presented with IAH, with a median Clarke test score of 4 and an interquartile range (IQR) of 4–6. The average RS scores for patients with T1D‐NAH and T1D‐IAH were 27.4 (IQR 2.85) and 27.65 (IQR 2.2), respectively, with no statistically significant difference (*p* = 0.983). Additionally, there was a trend towards better GFS(%) in T1D‐NAH patients (99; IQR 2.75) compared to T1D‐IAH patients (95.5; IQR 5.85), though this difference was not statistically significant (*p* = 0.0884).

### 5.2 Neuropsychological Evaluation

The scores obtained in the neuropsychological tests and its classification in normal, limit or abnormal are summarised at Table 2.

**TABLE 2:** Neuropsychological tests results and their corresponding qualitative results according to Neuronorma [18].

Neuropsychological test	Score	Normal	Limit	Altered
TAM	46 (44–48)	27 (90%)		3 (10%)
CERAD				
Memory	46 (44–48)	25 (83.33%)	3 (10%)	2 (6.67%)
Recall	6 (5–7)	20 (66.67%)	8 (26.67%)	2 (6.67%)
Recognition	20 (20–20)	23 (76.67%)	7 (23.33%)	0
TMT‐A^a^	8 (7–10)	19 (63.33%)	6 (20%)	5 (16.67%)
TMT‐B^a^	8.5 (7–10)	19 (63.33%)	6 (20%)	5 (16.67%)
Phonetic verbal fluency^a^	9 (8–11)	24 (80%)	4 (13.33%)	2 (6.67%)
Semantic verbal fluency^a^	9 (7–10)	21 (70%)	3 (10%)	6 (20%)
Boston naming test^a^	10 (8–13)	25 (83.33%)	5 (16.67%)	0

Abbreviations: CERAD: Consortium to Establish a Registry for Alzheimer's Disease; TAM: memory alteration test; TMT: trail making test.

^a^ Corrected standardised score according to NEURONORMA [18].

**FIGURE 1:**
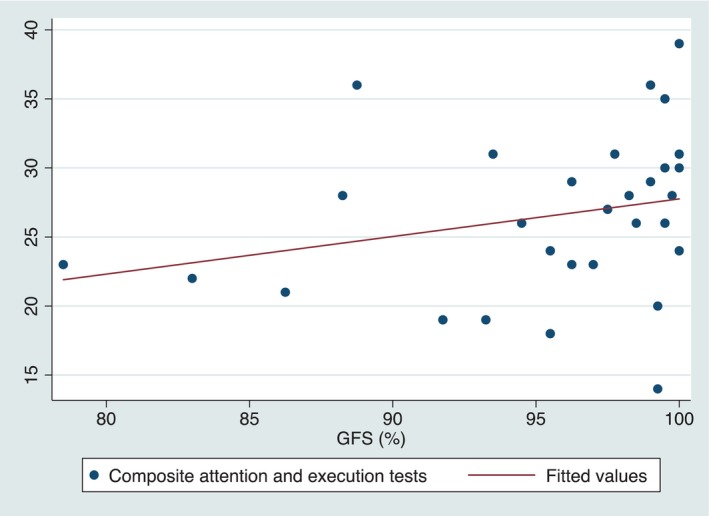
Scatter plot with line of best fit between gaze fixation stability (GFS) and the composite of executive and attention tests. The higher the GFS, the better the higher the scores in executive and attention tests.

A positive statistically significant correlations were found between RS and TAM (rho 0.43, *p* = 0.0171), and between RS and CERAD recall and recognition (rho 0.53 and 0.4, *p* = 0.002 and 0.027, respectively). A trend was also found between RS and CERAD memory (rho 0.31, *p* = 0.090). When adjusting for age in a multiple regression model, the significance of the correlation between SR and TAM was maintained (*p* = 0.016), while for CERAD recall, recognition and memory, the correlations became non‐statistically significant (*p* = 0.205, 0.124 and 0.24, respectively). When assessing the correlation with GFS, a positive significant correlation was found between GFS(%) and a composite of executive and attention tests (TMT‐A, TMT‐B, and phonemic verbal fluency) (rho 0.387, *p* = 0.0344), which remained unchanged when adjusting for age (*p* = 0.025) (shown in Figure 1). When using BCEA to assess fixation, we found a negative correlation with both BCEA63 (rho −0.397) and BCEA95 (rho −0.375), being both correlations statistically significant (*p* = 0.029 and 0.041, respectively). Both correlations remained unchanged when adjusting for age (*p* = 0.015 and 0.016, respectively). No statistically significant correlations were found with semantic verbal fluency.

### 5.3 NfL Concentrations

Median plasma NfL concentrations were of 10.95 pg/mL (9–15.93). A negative correlation between RS and plasma NfL concentrations was observed (rho −0,415, *p* = 0.0283). After adjusting by age, this correlation became non‐significant (*p* = 0.829).

### 5.4 CGM‐Derived Glucometric Parameters

We evaluated the CGM‐derived parameters and its relationship with MPR. Two patients were excluded as they did not use any type of CGM, and five patients were excluded due to having a time of use of the device below 80%. CGM‐derived data are shown in Table 3.

**TABLE 3:** Continuous glucose monitoring derived parameters^a^ and both retinal sensitivity and gaze fixation stability.

*n*	23
Age (years)	55 ± 8
Sex (% females)	43
T1D duration (years)	31.33 ± 9.27
Insulin therapy (%)	
Multiple daily injections	69.57
Insulin pump	30.43
CGM device (%)	
FreeStyle Libre 2	83.33
Guardian 3	8.7
Dexcom G6	4.17
Time of use (%)	99 (93–100)
TAR (%)	32 (23–43)
TIR (%)	62 (57–74)
TBR (%)	4 (2–6)
GV (%)	36.5 (33.2–39.2)
Number of hypoglycaemic events	57.5 (26–79)
GMI (%)	7 (6.8–7.5)
RS (dB)	27.75 (27.13–28.53)
GFS (%)	98.38 (94–99.5)

Abbreviations: CGM, continuous glucose monitor; GFS, gaze fixation stability; GMI, glucose management indicator; GV, glucose variability; RS, retinal sensitivity; T1D, Type 1 diabetes; TAR, time above range; TBR, time below range; TIR, time in range.

^a^ Data obtained from the last 90 days.

**FIGURE 2:**
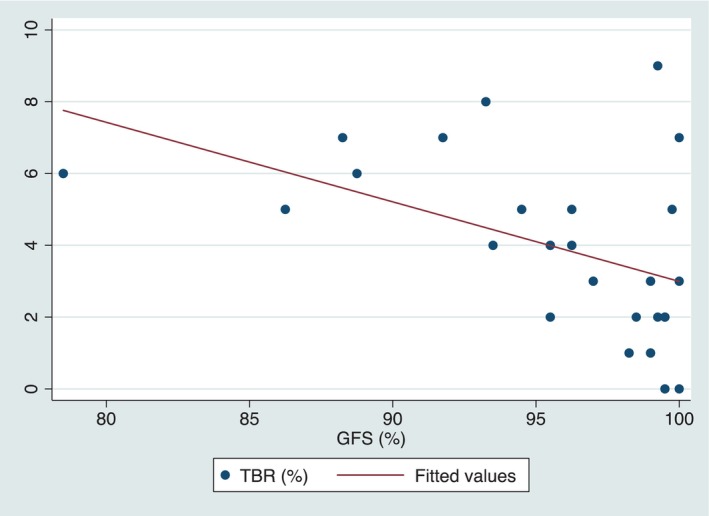
Scatter plot with line of best fit between gaze fixation stability (GFS) and time below range (TBR). The higher the TBR, the worse the GFS.

A statistically significant negative correlation between GFS(%) and TBR was found (rho −0.442, *p* = 0.034) (see Figure 2), indicating that people with a higher TBR showed worse GFS. This correlation remained significant after adjusting by age (*p* = 0.037). When assessing fixation through BCEA, positive correlations were found with TBR (BCEA63: rho 0.457, *p* = 0.019; BCEA95: rho 0.443, *p* = 0.023), remaining statistically significant after adjusting for age (*p* = 0.044 and 0.046, respectively). No correlations were found between MPR and the other glucometric parameters.

## 6 Discussion

This pilot study represents the first attempt to evaluate the potential usefulness of MPR as an early diagnostic tool for subclinical cognitive dysfunction and brain damage in individuals with T1D. Our results showed: (1) A significant correlation (adjusted for age) between RS and memory alteration tests (2) A significant correlation between gaze fixation and a composite of attention and executive neuropsychological tests; (3) A significant correlation between gaze fixation and time below range. No correlation was found between MPR and NfL. When comparing T1D‐IAH and T1D‐NAH individuals, we only observed a tendency for better GFS in T1D‐NAH.

The significant correlations observed between MPR measures and cognitive performance in individuals with T1D provide valuable insights into the potential utility of MPR as a tool for assessing cognitive function. The correlation between RS and memory alteration test (TAM) suggests that retinal function, as measured by MPR, may serve as a potential biomarker for memory impairment in individuals with T1D. The retina, composed of specialised cells that detect light and transmit visual information to the brain, has been increasingly recognised as an extension of the central nervous system. The positive correlation between RS and memory performance supports the notion that retinal health and function may reflect broader neurodegenerative processes affecting memory‐related brain regions, thus serving as a biomarker for cognitive dysfunction.

Furthermore, the correlation between GFS(%) and a composite of attention and executive neuropsychological tests highlights the interplay between cognitive dysfunction in attention and executive areas and GFS. GFS is a measure of an individual's ability to maintain focused visual attention, and its association with attention and executive functioning suggests that impaired cognitive function in these domains may contribute to disturbances in GFS. In other words, our results suggest cognitive dysfunction leading to poorer attention and executive performances also leads to worse GFS in people with T1D. These findings underscore the intricate relationship between visual attention and higher‐order cognitive processes. Thus, GFS and BCEA (BCEA63 and BCEA95) could also be used as a biomarker for cognitive dysfunction.

Although NfL values of normality are not well established, its concentration has shown to be a robust biomarker in some types of dementia [11, 25]. Moreover, in people with T1D‐IAH [10], NfL levels correlate with structural brain damage. In the present study, NfL plasma concentration did not show a significant association with MPR data, possibly due to the small number of subjects included and the absence of a clinically meaningful cognitive impairment.

Additionally, our data indicate that individuals with a greater TBR present with worse GFS. This could suggest that a greater exposure to hypoglycaemia could influence MPR outcomes, further underscoring the importance of early monitoring and intervention in this population [1, 26]. However, a causal effect cannot be inferred due to the design of the study. In our study we assessed the number of severe hypoglycaemic events from the past 5 years, only finding a trend of negative correlation with GFS, but we do not know the total number of events in the duration of the disease, and the use of continuous glucose monitoring with alarm systems in the latest years may have contributed to a reduction of hypoglycaemic events. All this supports a possible role of hypoglycaemia in brain damage, although further studies are needed.

The main strong points of our work are the following: (1) It is the first study to evaluate MPR in T1D as a potential tool for early diagnostic of cognitive dysfunction; (2) The results confirm that MPR could become a quick (∼20 min), easy, non‐invasive, reproducible and inexpensive method to evaluate early cognitive alterations, in contrast to invasive, costly and/or time‐consuming methods (MRI, neurocognitive evaluation tests, etc).

Despite these promising results, some limitations of this study should be noted. First, our sample size was relatively small, limiting the generalizability of our findings. Additionally, we did not include any participants with clinical cognitive impairment, which limits the ability to draw conclusions about the sensitivity and specificity of MPR as a diagnostic tool in this population. The variability of subclinical cognitive dysfunction detected in the neuropsychological tests was low, which limits the evaluation of another technique (MPR) to serve as a diagnostic tool. Moreover, most of our participants fell within the normal range for MPR results. It should be noted that in our previous studies MRI and neuropsychological measures showed difference between T1D‐IAH and T1D‐NAH, but MPR did not. One hypothesis could be that these specific measures show changed distinct from one resulting from hypoglycaemia; another could be that the current study was underpowered as we had to exclude 10 participants and variability of both cognitive dysfunction and MPR was low. Both retinopathy and glaucoma independently affect the outcome of MPR which limits the applicability of this technique in this population.

To make a more solid conclusion about the utility of MPR it would seem prudent to conduct a larger study with more participants with T1D with a broader spectrum of duration of diabetes, clinical cognitive impairment, and perhaps using age‐based controls without diabetes, as the absence of a control group limits the capacity to accurately assess the specific impact of diabetes on cognitive function and differentiate it from normal age‐related cognitive changes. Moreover, studies including people with different degrees of retinopathy would allow us to determine if MPR can be applicable in this population.

## 7 Conclusions

In this first exploratory study, we evaluated the utility of MPR in detecting early cognitive dysfunction and brain damage in individuals with T1D. Our results demonstrate that MPR is a promising, non‐invasive, easy‐to‐perform, and inexpensive diagnostic tool for assessing early subclinical cognitive dysfunction. Our findings also show a link between greater exposure to hypoglycaemia and worse GFS, indicating a possible influence of hypoglycaemia on MPR outcomes, independently of hypoglycaemia awareness in our study population. However, due to study limitations, further research is needed to clarify this relationship and its impact on brain function.

More research is needed to reproduce and extend these findings in larger and more diverse populations. Including patients with clinical cognitive impairment in future studies will help assess the sensitivity and specificity of MPR as a diagnostic tool.

## Author Contributions

**Manel Mateu‐Salat:** conceptualization, methodology, formal analysis, investigation, resources, writing – original draft, visualization, and funding acquisition. **Nicole Stanton‐Yonge:** conceptualization, investigation, resources and writing – review and editing. **Frederic Sampedro Santaló:** software, formal analysis, writing – review and editing. **José Ignacio Vela:** conceptualization, investigation, resources. **Jesús Díaz Cascajosa:** conceptualization, investigation, resources. **Eva Safont Pérez:** investigation. **Daniela Rego‐Lorca:** writing – review and editing. **Ana Chico:** conceptualization, methodology, resources, writing – review and editing, supervision, and funding acquisition.

## Ethics Statement

The study was approved by the Institutional Review Board of Institut de Recerca Sant Pau and all participants gave written informed consent to participate. All procedures performed in studies involving human participants were in accordance with the ethical standards of the institutional research committee and with the 1964 Helsinki declaration and its later amendments or comparable ethical standards.

## Conflicts of Interest

The authors declare no conflicts of interest.

## Data Availability

The data that support the findings of this study are available from the corresponding author upon reasonable request.
